# Design and Implementation of Low Profile Antenna for Dual-Band Applications Using Rotated E-Shaped Conductor-Backed Plane 

**DOI:** 10.1155/2014/632403

**Published:** 2014-02-23

**Authors:** Mahdi Jalali, Tohid Sedghi, Shahin Shafei

**Affiliations:** ^1^Department of Electrical Engineering, Naghadeh Branch, Islamic Azad University, Naghadeh, Iran; ^2^Department of Electrical Engineering, Urmia Branch, Islamic Azad University, Urmia, Iran; ^3^Young Researchers and Elite Club, Mahabad Branch, Islamic Azad University, Mahabad, Iran

## Abstract

A novel configuration of a printed monopole antenna with a very compact size for satisfying WLAN operations at the 5.2/5.8 GHz and also for X-band operations at the 10 GHz has been proposed. The antenna includes a simple square-shaped patch as the radiator, the rotated U-shaped conductor back plane element with embedded strip on it, and the partial rectangular ground surface. By using the rotated U-shaped conductor-backed plane with proper values, good impedance matching and improvement in bandwidth can be achieved, at the lower and upper bands. The impedance bandwidth for *S*_11_ < −10 dB is about 1.15 GHz for 5 GHz band and 5.3 GHz for X-band. The measured peak gains are about 1.9 dBi at WLAN-band and 4.2 dBi at X-band. The experimental results represent that the realized antenna with good omnidirectional radiation characteristics, enough impedance bandwidth, and reasonable gains can be appropriate for various applications of the future developed technologies and handheld devices.

## 1. Introduction

There is a prodigious increase in the applications that utilize wireless local area network technology and the high data rate communications which has resulted in wireless devices expected to function under different multiband environments. An exclusively impellent issue for these applications is the design and development of multiband antennas which are simple structures and possess multiband functionality. The best candidate for implementation of multiband antennas is on planar technology as it allows easy integration with microwave integrated circuits and is lightweight and relatively low cost [1–7]. Multiband technology requires compact antennas for mobile and wireless communication systems. Low profile and miniaturized antennas have unwanted intrinsic attribute, for instance, inappropriate radiation characteristics. Furthermore miniaturization is particularly considerable for the antenna designer and arises out of the limited available volume of the wireless handset casing [4–10]. Printed monopole antenna (PMA) exhibits principal parameters in designing multiband antennas such as agreeable radiation patterns all over the designated operational bands, ease of fabrication, and compact size [8–10]. Several designs in the literature concerning the PMA with multiband specifications and large size or even small size have been reported lately. Those designs use different forms of conductor-backed plane in bottom layer of substrate, parasitic elements around the radiator, for exciting dual or multifrequency mode [7–9]. The disadvantages of these antennas include the inefficient broadband matching, complexity to realize the required operating frequency bands, and unacceptable gain.

In this paper, a novel microstrip-fed monopole antenna is constructed and tested to cover the following operational bands: WLAN (5150–5350/5725–5825 MHz specified by IEEE 802.11a) and X-band (8–12 GHz). In this antenna, the aim is to propose a simple configuration of patch and ground plane with a step-by-step process design of conductor back plane to access dual-band specification. Frequency bands can be created, by inserting the rotated U-shaped conductor-backed plane, and for proper controlling the both bands, the strip is embedded on them. The impedance matching of the antenna is enhanced by tuning and suitable adjusting dimensions of inserted strip. Wider impedance bandwidth is obtained by using of final structure of conductor-backed plane, which provides a wide usable fractional bandwidth 21% for 5 GHz WLAN and 52% for X-band. The realized antenna has simple structure and compact size of 15 × 15 mm^2^; moreover, it can provide good omnidirectional radiation patterns. Measured and simulated results of the antenna with the different forms of conductor-backed plane are presented.

## 2. Monopole Antenna Design

**Figure 1:**
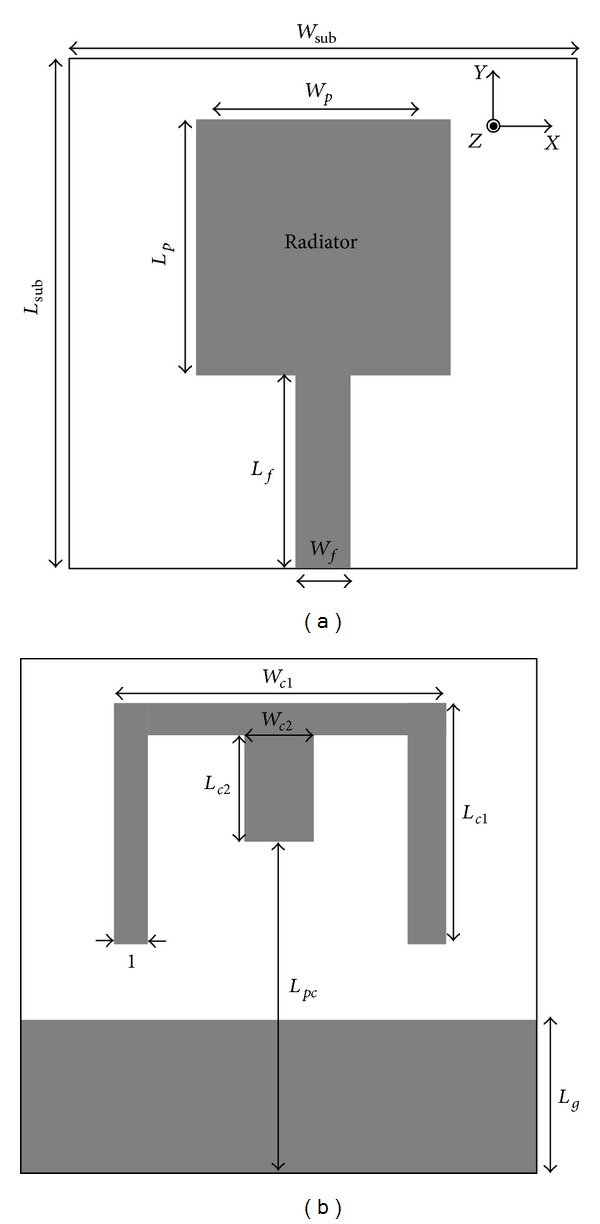
Geometry of the proposed antenna. (a) Squared-shaped patch. (b) Rotated U-shaped conductor back plane with strip attached on it, rectangular-shaped ground plane. Dimensions (units: mm).

**Figure 2:**
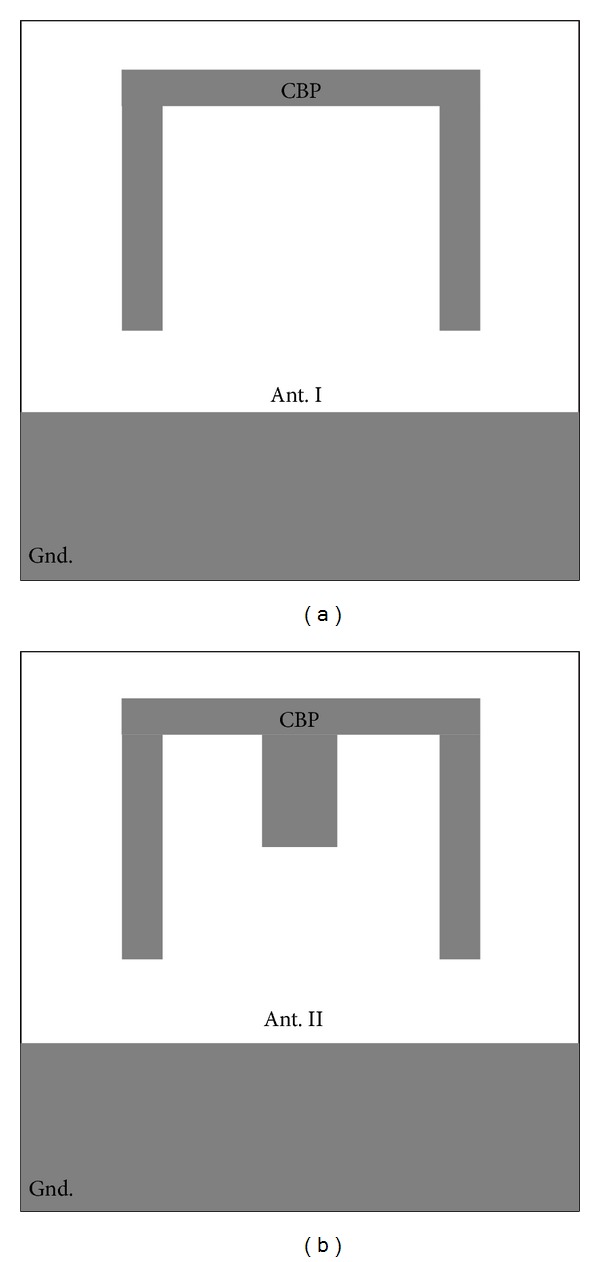
Configurations of conductor-backed plane. (a) Ant. I (b) Ant. II (conductor-backed plane: CBP).

The configuration of the proposed antenna with *L*_sub_ × *W*_sub_ dimensions is illustrated in Figure 1. The top layer includes the main radiator in the form of square patch with the dimensions of 7.5 × 7.5 mm^2^. The bottom layer contains rectangular-shaped ground plane and conductor-backed plane. The proposed antenna, with compact dimensions of 15 × 15 mm^2^, is fabricated with a substrate made of FR4, with the thickness 0.8 mm and the relative dielectric constant 4.4 and loss tangent 0.024. The width of the feed-line microstrip is fixed at 1.6 mm for 50-Ω impedance. For the impedance matching, the distance between the radiator and the ground plane is indicated with a gap (gap = *L*_*f*_ − *L*_*g*_), which provides proper control between the lower edge patch and the ground plane. The optimum gap between the radiator and the ground plane is 1.2 mm. To modify the performance of the antenna for creating two bands WLAN and X-band, the conductor-backed plane is designed in the manner shown in Figure 2. The conductor-backed plane is designed in two steps:

 Step (I): create a rotated U-shaped conductor-backed plane (Figure 2(a));
 Step (II): insert a strip to previous step (Figure 2(b)).

With inserting rotated U-shaped conductor-backed plane, band of 8–12 GHz (X-band) operation can be achieved, whereas, by using strip inside the conductor-backed plane, 5.25–5.85 GHz (WLAN) can be achieved and also additional resonance frequency is generated. The conductor-backed dimensions are optimized by parametric study to obtain enhanced impedance matching over the frequency bands. In the design process, the dimensions of the structure are optimized using the software HFSS for multiple-frequency coverage.

The optimal parameters of the constructed antenna are as follows: *W*_sub_ = 15 mm, *L*_sub_ = 15 mm, *W*_*p*_ = 7.5 mm, *L*_*p*_ = 7.5 mm, *L*_*f*_ = 5.7 mm, *W*_*f*_ = 1.6 mm, *L*_pc_ = 9.3 mm, *W*_*c*1_ = 9.6 mm, *L*_*c*1_ = 7 mm, *W*_*c*2_ = 2 mm, *L*_*c*2_ = 3 mm, and *L*_*g*_ = 4.5 mm. The other optimized dimensions of the antenna are indicated in Figure 1.

## 3. Simulation and Measurement Result

The designed antenna was fabricated and measured. The numerical and simulation results are achieved using the software Ansoft HFSS. The impedance bandwidth of the constructed antenna was tested by Agilent 8722ES Vector Network Analyzer in the Antenna Measurement Laboratory at Iran Telecommunication Research Center. While measuring the characteristics of realized antenna, some problems appear:

1. changing the input impedance of the antenna,
2. distortion of the far-field radiation pattern.

**Figure 3:**
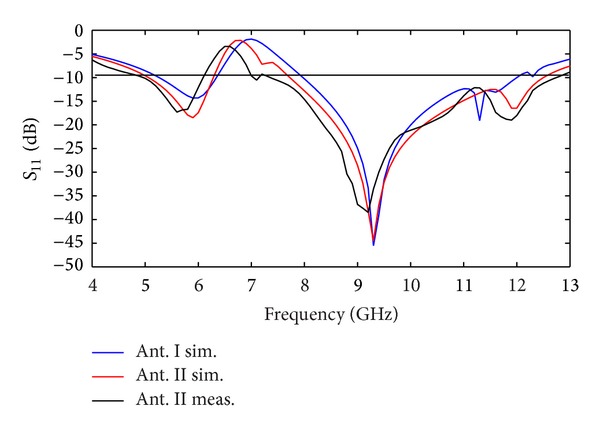
Measured and simulated *S*_11_ of the proposed dual-band operation antennas.

**Figure 4:**
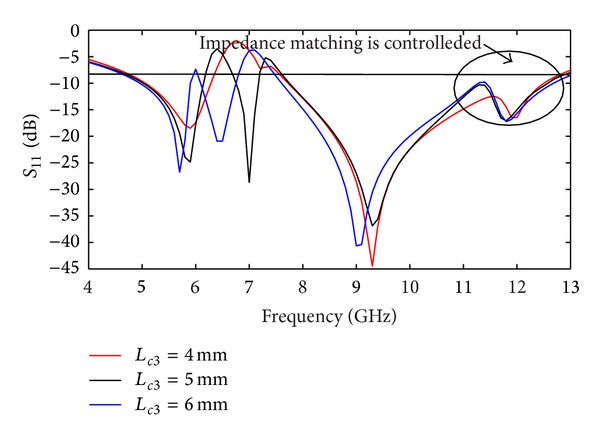
Simulated *S*_11_ for different values of *L*_c2_ when *W*_c2_ is 2 mm.

**Figure 5:**
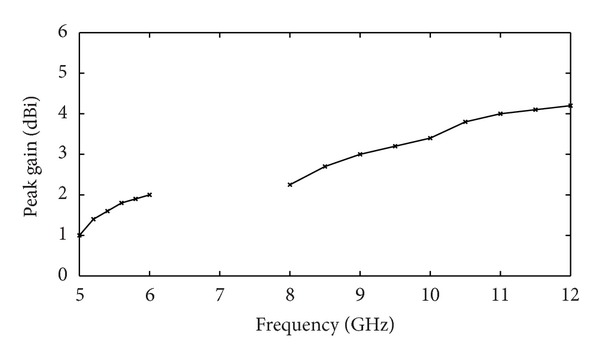
Measured antenna peak gain for Ant. II.

**Figure 6:**
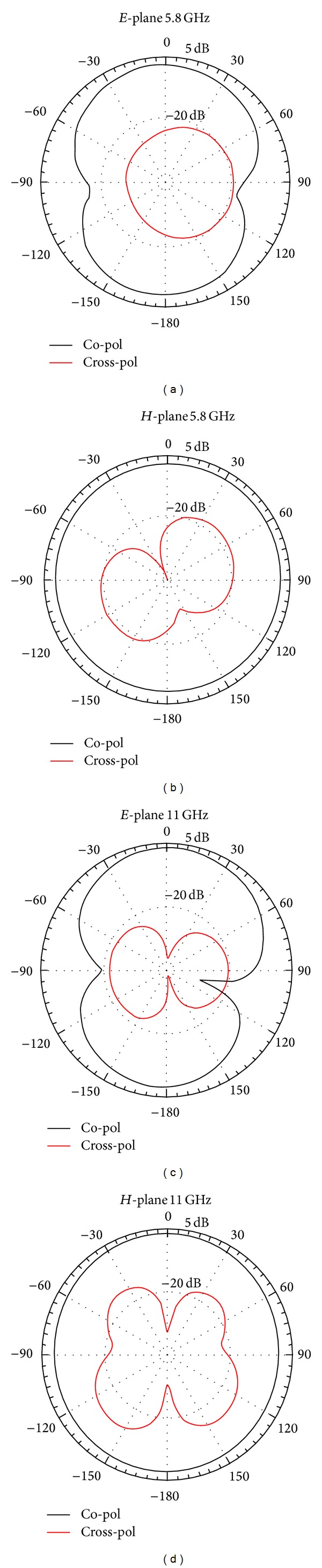
The radiation pattern of the proposed antenna at different frequencies, 5.8 GHz and 11 GHz.

These problems emerge because the coaxial cable is not connected to the antenna properly and it becomes part of the radiating structure. Several methods were presented in the literature [10, 11], for solving those problems, such as using high-impedance ferrite beads along the cable near to its connection to absorb the induced power or utilizing optic links. Figure 2 shows the structures of the various conductor-backed planes. With introducing, initial rotated U-shaped conductor-backed plane in the manner indicated in Figure 2(a), the X-band can be achieved as shown in Figure 3. By embedding strip in the manner shown in Figure 2(b), on the conductor-backed plane, 5 GHz WLAN band can be obtained as depicted in Figure 3. Also, it is perceived from Figure 3 that a proper control on the dual-band operation can be obtained when strip is attached. The simulated and measured *S*_11_ characteristic of the proposed antenna is depicted in Figure 3. This figure shows there is generally good correlation between the measurement and simulation results. Furthermore, Figure 3 clearly shows that the measured impedance bandwidth of Ant. II very well covers the dual-band characteristics in 5–6.15 GHz and 7.5–12.8 GHz for *S*_11_ < −10 dB. The effect of strip element on the antenna's performance is investigated in Figure 4. It can be observed that, by choosing a suitable size for the strip (*L*_c2_), impedance bandwidth of lower band and the impedance matching of upper band are improved and much wider impedance bandwidth can be produced. The measured peak gain from 5-6 GHz (W-LAN) and 8–12 GHz (X-band) is plotted in Figure 5. This figure highlights that the realized antenna has good gain flatness at the WLAN band (gain variation of less than 1 dB), whereas antenna gain is increased dramatically from about 2.1 to 4.2 dBi for X-band.  Figure 6 shows the measured radiation pattern for four different frequencies of operating bands at 5.8 GHz and 11 GHz in H-plane and E-plane. The figure approximately exhibits an omnidirectional radiation pattern in H-plane and a monopole-like radiation pattern in the E-plane. From an overall view of these radiation patterns, the proposed antenna behaves quite similarly to the typical printed monopoles in the operating bands.

## 4. Conclusion

A small and novel microstrip-fed monopole antenna is proposed and fabricated for multiband wireless communication systems and mobile devices. The main features of the proposed antenna are the compact dimensions and dual-band performances that are achieved without modifying the ground plane or radiator. Multiband characteristics are obtained, by embedding the rotated U-shaped conductor-backed plane. For controlling the desired bands, the strip size is tuned by parametric study. The effect of the U-shaped strips dimensions on the optimization of the impedance matching is discussed in detail. Also, the proposed antenna has a compact size of 15 mm × 15 mm × 0.8 mm and is measured to cover the bandwidth for IEEE 802.11a and also X-band. Good omnidirectional radiation pattern characteristics and enough gains are obtained over the operating bands.
